# Polysaccharides from sea buckthorn — Ultrasound-assisted enzymatic extraction, purification, structural characterization, and antioxidant activity analysis

**DOI:** 10.1016/j.fochx.2025.102265

**Published:** 2025-02-05

**Authors:** Jing Wen, Ruijie Huang, Shi Li, Lin Jiang, Liheng Shao, Qin Zhang, Chunhui Shan

**Affiliations:** a Engineering Research Center of Storage and Processing of Xinjiang Characteristic Fruits and Vegetables, Ministry of Education, School of Food Science, Shihezi University, Shihezi, Xinjiang 832000, China; b Key Laboratory of Processing and Quality and Safety Control of Specialty Agricultural Products (Co-construction by Ministry and Province), Ministry of Agriculture and Rural Affairs, School of Food Science, Shihezi University, Shihezi, Xinjiang 832000, China; c Key Laboratory for Food Nutrition and Safety Control of Xinjiang Production and Construction CorpsSchool of Food Science, Shihezi University, Shihezi, Xinjiang 832000, China

**Keywords:** Polysaccharide, Ultrasound-assisted enzyme extraction, Sea buckthorn, Antioxidant activity

## Abstract

This study employed a sophisticated approach consisting of ultrasound-assisted enzyme treatment to extract polysaccharides from sea buckthorn (SBP). The SBP extraction parameters were optimized, the following optimal parameters were identified: solid–liquid ratio of 1:32 g/mL, ultrasound duration of 26 min, ultrasound temperature of 52 °C, and composite enzyme concentration of 6000 U/100 mL, and the maximum extraction yield of SBP was 24.07 ± 0.15 %. The separation and purification of SBP resulted in the isolation of three fractions of polysaccharides (SBPR-1, SBPR-2, SBPR-3). The composition and structural characteristics of the SBPRs were identified. Furthermore, the SBPRs exhibited the characteristic absorption peaks of polysaccharides. Notably, the surface microstructures of the SBPRs showed significant variations. Moreover, all SBPRs demonstrated commendable thermal stability and in vitro antioxidant activity. This study serves as a reference for the development and application of natural antioxidants and provides a theoretical foundation for the environmentally friendly and effective extraction of SBP.

## Highlights

• Sea buckthorn polysaccharide (SBP) was extracted by ultrasound-assisted enzyme.
• Three polysaccharide fractions (SBPR-1, SBPR-2, and SBPR-3) were purified.
• The physicochemical and structural characteristics of SBPRs were evaluated.
• SBPRs exhibit excellent thermal stability and antioxidant activity.

## 1 Introduction

Sea buckthorn (*Hippophae rhamnoides* L.), also referred to as blackthorn and vinegar willow, is the fruit of a deciduous shrub belonging to the family Elaeagnaceae. China accounts for 90 % of the world's sea buckthorn cultivation, and is thusthe global leader in sea buckthorn resources and reserves. In China, sea buckthorn is extensively cultivated across 19 provinces, such as Xinjiang, Qinghai, and Gansu, typically thriving at altitudes between 800 and 3600 m (Kurskaya et al., 2022). Notably, sea buckthorn was incorporated into the “Chinese Pharmacopoeia” in 1977. As a plant with both its medicinal and culinary applications, sea buckthorn possesses significant health benefits. It is rich in a variety of nutrients and bioactive compounds, including polysaccharides, vitamins, flavonoids, steroids, amino acids, and organic acids. Owing to the presence of these compounds, sea buckthorn is used in traditional medicine for invigorating the spleen, removing phlegm, dispersing blood stasis, and promoting blood circulation (Wang, Zou, et al., 2024). Currently, sea buckthorn is widely utilized in functional foods, pharmaceuticals, cosmetics, and animal feed. Xinjiang — with its vast desert and Gobi landscapes — provides optimal growth conditions for sea buckthorn and thus has the largest sea buckthorn resources in China. The sea buckthorn grown in this region has a significantly higher bioactive content and offers greater functional benefits compared to the sea buckthorn cultivated in the plains (Zhu et al., 2022).

Polysaccharides are among the most crucial active constituents of sea buckthorn, exhibiting exceptional physicochemical and biological properties, including immunomodulation (Tang, Wang, Chen, et al., 2023), anti-inflammatory effects (Han et al., 2021), antioxidation (Kubczak et al., 2022), glycemic regulation (Yang et al., 2024), and hepatoprotection (Wang et al., 2018). Sea buckthorn polysaccharides (SBP) are used as natural food seasonings and additives, serving as natural sweeteners, colorants, and flavor enhancers. Additionally, SBP can act as natural antioxidants, enabling the development of novel functional foods and pharmaceuticals. So far, studies on the active components of sea buckthorn have primarily focused on its flavonoid composition, with relatively limited research on SBP.

Effective extraction methods are essential for studying the structure and biological activity of polysaccharides (Ling et al., 2024). Although the hot water extraction technique remains the predominant method for extracting plant polysaccharides, it requires high extraction temperatures and long extraction durations. Moreover, it is energy-intensive, and provides low extraction rates (Ling et al., 2024). Thus, to improve the efficiency and environmental safety of extraction processes, researchers are increasingly exploring innovative techniques, such as ultrasound, microwave, and enzyme-assisted extraction and combining them to achieve synergistic effects. Studies have already demonstrated that combined treatment methods can significantly enhance the extraction of plant polysaccharides (Zhang et al., 2020). Of these methods, ultrasound-assisted enzymatic extraction effectively combines the advantages of ultrasound and enzyme-assisted extraction techniques and thus represents a particularly promising approach. This method is characterized by its convenient operation, high yield, low energy consumption, mild reaction conditions, and environmental safety (Li et al., 2023; Ling et al., 2024). By combining ultrasonic cavitation with the enzyme-assisted hydrolysis of the cell wall, active ingredients are more efficiently released, resulting in a higher extraction rate (Fu, Wang, Belwal, Xie, et al., 2021). Polysaccharides from numerous plants, including *Ulva Lactuca* (Wang, Li, et al., 2024), *Tremella fuciformis* (Hou et al., 2024), and *Rosa roxburghii*(Jiao et al., 2024), have been extracted using this technique. Nevertheless, how the application of this technique affects the monosaccharide composition, structural conformation, and antioxidant properties of SBP is yet to be studied in detail.

In this study, sea buckthorn berries sourced from Xinjiang were used to obtain polysaccharides through an ultrasound-assisted enzymatic extraction method. The response surface approach was applied to optimize the extraction process and identify the most suitable extraction conditions. To obtain purified polysaccharide components, SBP separation and refinement were performed using DEAE-52 cellulose and Sephadex G-150 columns. The polysaccharide components were then evaluated, and the differences in their chemical constituents (including total sugar, protein, and uronic acid content), monosaccharide composition, and preliminary structural characteristics were examined. Finally, the in vitro antioxidant capacities of these polysaccharides were investigated. The present study provides a scientific foundation for the effective extraction and utilization of SBP.

## 2 Materials and methods

### 2.1 Reagents and materials

Sea buckthorn was obtained from Korla, Xinjiang, China. The sea buckthorn berries were ground and freeze-dried before defatting with petroleum ether. After processing, the material was crushed, passed through a 40-mesh sieve, packaged, and stored at a low temperature for subsequent use. Cellulase (50 U/mg) and pectinase (30 U/mg) were supplied by Shanghai Macklin Biochemical Technology Co., Ltd. Meanwhile, Shanghai SolarBio Bioscience & Technology Co., Ltd. provided DEAE cellulose-52 and Sephadex G-150. Every reagent used in this study was of analytical grade and was purchased from a commercial supplier.

### 2.2 Extraction of SBP

#### 2.2.1 Ultrasound-assisted enzymatic extraction of SBP

First, 1.00 g of sea buckthorn powder was weighed. Deionized water was added in accordance with the required solid–liquid ratio (1:30 g/mL), and the pH of the solution was adjusted to 5. For enzymatic hydrolysis, a composite enzyme solution (cellulase and pectinase at a 1:1 U/100 mL ratio) was added, and the mixture was incubated in a water bath at 50 °C. Once the hydrolysis reaction was complete, the enzymes were inactivated by raising the temperature to 80 °C for 15 min. Subsequently, ultrasonic extraction was performed at a power of 400 W and temperature of 45 °C for 25 min. Once the extraction was complete, the mixture was centrifuged at 6000 rpm for 20 min to collect the supernatant. The polysaccharide yield (%) was calculated using formula (1).

(1) $$\text{Yield of}\;\mathrm{SBP}\;\left(\%\right)=\frac{\text{Weight of crude polysaccharides}\;\left(g\right)}{\text{Weight of sample}\;\left(g\right)}\times 100\%$$

The supernatant was concentrated and proteins were removed using the Sevag method. Following dialysis, the concentrate was precipitated in four times the volume of absolute ethanol at 4 °C for 24 h. Centrifugation was used to collect the precipitate, which was successively cleaned with acetone and anhydrous ethanol. Finally, crude SBP were obtained by vacuum freeze-drying the precipitate.

#### 2.2.2 Single-factor optimization experiments

For process optimization, the effects of six parameters (solid–liquid ratio, ultrasound duration, ultrasound temperature, amount of enzyme, hydrolysis time, and ultrasound power) on SBP yield were examined based on the extraction technique described in section 2.2.1. Single-factor experiments were conducted using the following conditions: solid–liquid ratio, 1:20–1:40 g/mL; ultrasound duration, 15–35 min; temperature, 25 °C–65 °C; addition of composite enzyme, i.e., cellulase and pectinase at a 1:1 ratio, 2000–10,000 U/100 mL; hydrolysis time 70–110 min; and ultrasound power, 280–560 W.

#### 2.2.3 Response surface test design

Based on the single-factor experiments, an optimization study was conducted using the Box-Behnken response surface approach. Ideal SBP extraction parameters were identified using Design-Expert 13 software. The SBP extraction rate was added as the response variable in the response surface experiment, with the four independent variables being the compound enzyme amount (D), ultrasound temperature (C), ultrasonic duration (B), and solid–liquid ratio (A). The level design is presented in Table 1.

**Table 1:** Box-Behnken response surface methodology.

Levels	Variables			
	A: Solid-liquid ratio (g/mL)	B: Ultrasound time (min)	C: ultrasound temperature (°C)	D: compound enzyme amount (U/100 mL)
-1	1:25	20	35	4000
0	1:30	25	45	6000
1	1:35	30	55	8000

### 2.3 SBP purification

SBP purification was performed as described previously, with minor modifications (Wei & Zhang, 2023; Yang et al., 2023). Sephadex G-150 gel chromatography columns and DEAE-52 cellulose resin were used for purification procedures. A polysaccharide solution (80 mg/mL) was prepared, and insoluble elements were removed via centrifugation. The crude polysaccharides were separated using an anion exchange column packed with DEAE-52 cellulose (1.6 cm × 60 cm). Gradient elution was performed using deionized water, followed by 0.2, 0.4, 0.6, and 0.8 mol/L NaCl. The flow rate was set to 1 mL/min, and an automatic collector (8 mL/tube) was employed to collect SBP fractions. The NaCl eluates were concentrated and dialyzed before freeze-drying to obtain the preliminary purified SBP fractions. The UV absorbance of the eluate in every alternate tube was measured at 490 nm using the phenol‑sulfuric acid method. Further purification was performed on a Sephadex G-150 gel column (3.5 cm × 60 cm). Each polysaccharide fraction was completely dissolved in 5 mL of deionized water, and insoluble materials were removed via centrifugation. Distilled water was used for elution at a flow rate of 0.2 mL/min (8 mL/tube). The phenol‑sulfuric acid assay was employed, and the UV absorbance of the eluate in each tube was detected at 490 nm. To acquire the pure SBP fractions SBPR-1, SBPR-2, and SBPR-3, the eluates were collected and lyophilized.

### 2.4 Chemical composition of SBPRs

The phenol‑sulfuric acid method was employed to determine the total sugar concentration in the SBPRs, with glucose serving as the reference (Tang, Wang, Huang, & Huang, 2023). Meanwhile, using bovine serum albumin as the standard, the protein concentration of the SBPRs was determined via Coomassie Brilliant Blue staining (Jiao et al., 2024). Finally, the *M*-hydroxybiphenyl technique was employed to quantify the amount of uric acid in the SBPRs, with galacturonic acid serving as the standard (Xue et al., 2023).

### 2.5 Characterization of SBPRs

#### 2.5.1 Determination of monosaccharide composition

The monosaccharide composition of the SBPRs was analyzed using ion chromatography (Jiang et al., 2024). First, a 5 mg sample was added to an ampoule, mixed with 2 mL of 3 M trifluoroacetic acid (TFA), and hydrolyzed at 120 °C. After hydrolysis, the acid solution was evaporated to dryness under nitrogen. Next, 5 mL water was added, and the mixture was vortexed. Then, 100 μL of the mixture was removed and added to 900 μL of deionized water; this solution was centrifuged at 12,000 rpm for 5 min. Subsequently, 25 μL of the supernatant was removed to analyze the hydrolysis products using a Dionex ICS-5000+ ion chromatography system equipped with a Dionex Carbopac™ PA20 anion exchange column (3 mm, 150 mm) and a Dionex ED50A electrochemical detector. The mobile phase consisted of H_2_O, 15 mM NaOH, 15 mM NaOH, and 100 mM NaAc, which were used for elution at varying ratios and a flow rate of 0.3 mL/min.

#### 2.5.2 Molecular weight of SBPRs

The molecular weight of SBPRs was determined using high-performance gel permeation chromatography (HPGPC). First, 5 mg of each sample was weighed and dissolved in 1 mL of the mobile phase (0.05 M NaCl) to prepare a 5 mg/mL solution. These solution was vortexed and centrifuged at 12000 rpm for 10 min. The supernatant was aspirated, filtered through a 0.22-μm microporous filter membrane, and then transferred to a 1.8 mL injection vial. A 25 μL sample was used for further analysis on an LC-10 A HPLC system equipped with a BRT105–103-101 tandem gel column (8 × 300 mm) and RID-20 A differential detector. The flow rate of the mobile phase (0.05 M NaCl) was 0.7 mL/min, the column temperature was 40 °C, and the analysis duration was 70 min. lgMp-RT (Mp peak molecular weight) and lgMw-RT (Mw weight average molecular weight) values were determined based on the retention time of dextran standards (Dextran standards 1153, P5, P10, P20, P50, P100, P200, P400 and P800). A standard curve of lgMn-RT (average molecular weight of Mn number) was generated to calculate the molecular weight of the samples. The relative molecular weights of the three polysaccharide fractions were calculated by measuring the retention times of the SBPRs.

#### 2.5.3 Fourier transform infrared spectroscopy (FT-IR) analysis

The potassium bromide (KBr) tablet method was used. Appropriate amounts of dried SBPR samples were mixed with KBr powder in agate. The mixture was compressed into pellets using the KBr disk method. The infrared spectra were scanned in the 400–4000 cm^−1^ region using a Bruker Vertex 70 infrared spectrometer. In total, 32 scans were performed at a resolution of 4 cm^−1^ (Cui et al., 2018).

#### 2.5.4 Scanning electron microscopy (SEM) analysis

A small sample of each the SBPR was placed on a sample stage with copper tape. Following processing in the vacuum coating machine, the surface morphology of the samples was observed at 5 kV and magnifications of 2000×, 5000×, and 8000× using a scanning electron microscope (SU8000, Hitachi, Tokyo, Japan).

#### 2.5.5 Thermal stability analysis

The thermal stability of SBPRs was examined using a simultaneous thermal analyzer (STA449F3, NETZSCH, Germany) (Zhang, Li, et al., 2022). The temperature range was 25 °C to 800 °C, the nitrogen flow rate was 30 mL/min, and the heating rate was 10 °C/min.

### 2.6 Antioxidant activities of SBPRs

The DPPH, ABTS and hydroxyl free radical scavenging activities of the SBPRs were examined in accordance with the earlier studies, The radical scavenging rate (*I*, %) was calculated using eq. (2) (Cai et al., 2020; Fang et al., 2020; Tang, Wang, Chen, et al., 2023). The total reducing power of the SBPRs (I) was determined according to previously described protocols, using eq. (3) (Fang et al., 2020).

(2) $$I\;\left(\%\right)=\frac{A_{n0}-\left(A_{n}-A_{m}\right)}{A_{n0}}\times 100\%$$

Here, A_n_ is the absorbance of the sample; A_m_ is the absorbance of the sample containing methanol instead of the reagent; and A_n0_ is the absorbance of the blank control (deionized water instead of the sample).

(3) $$I\;\left(\%\right)=A_{s}-A_{0}$$

Here, A_S_ is the absorbance of the sample, and A_0_ is the absorbance of the blank control (deionized water instead of the sample).

### 2.7 Statistical analysis

Graphs were prepared using Origin 2019, and variance analysis was conducted with SPSS 26.0. Response surface processing was performed using Design-Expert 13. Three iterations were conducted for each experiment, and the results were represented as mean ± standard deviation (X ± S). Differences were deemed significant when *p* < 0.05 and very significant when *p* < 0.01.

## 3 Results and discussion

### 3.1 Influence of different ultrasound-assisted enzymatic extraction conditions on the yield of SBP

#### 3.1.1 Effect of the solid–liquid ratio

**Fig. 1:**
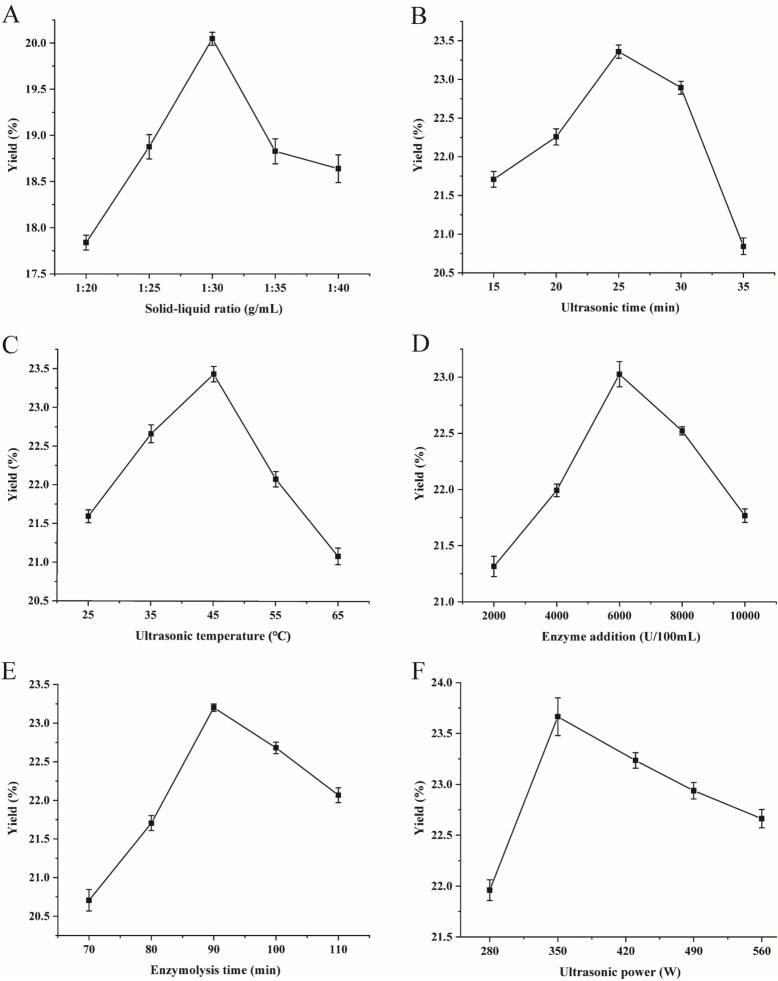
Single-factor experiments. Effects of the solid–liquid ratio (A), ultrasonic duration (B), ultrasonic temperature (C), compound enzyme concentration (D), hydrolysis time (E), and ultrasonic power (F) on the extraction yield of SBP.

The influence of the solid–liquid ratio on SBP extraction is shown in Fig. 1A. The yield of SBP increased gradually when the solid–liquid ratio changed from 1:20 to 1:30 (g/mL). As the amount of solvent increased, the viscosity of the system reduced, promoting the ultrasonic cavitation effect and the enzyme-induced breakdown of the cell wall. This enhanced the efficiency of mass transfer and permeability, increasing polysaccharide solubility (Xue et al., 2023). The highest polysaccharide extraction rate (20.05 ± 0.07 %) was obtained using a solid–liquid ratio of 1:30. However, when this ratio was gradually increased, the extraction rate showed a decline. This was potentially because the excessive solvent volume impeded the impact of ultrasonic waves on plant tissues, thereby diminishing their disruption capacity. Concurrently, the reduction in substrate concentration may also have affected the reaction rate of the compound enzyme (Lv et al., 2024; Zhang, Yang, et al., 2022). As a result, the optimal solid–liquid ratio for SBP extraction was determined to be 1:30 (g/mL).

#### 3.1.2 Effect of ultrasonic duration

During ultrasonic treatment, sound waves carry mechanical energy throughout the solvent, disrupting plant cells (Huang et al., 2022). The duration of ultrasonic treatment can thus influence the polysaccharide extraction rate. As shown in Fig. 1B, SBP extraction efficiency increased with the extension of the ultrasonic duration (15–25 min), peaking at 25 min (23.36 ± 0.09 %) gradually declining. This phenomenon could be attributed to the cumulative mechanical effects of ultrasonic waves on plant cells, causing a the significant disruption of cell walls and microbial matrices. Consequently, bioactive components were released into the solvent, leading to the observed peak in polysaccharide content. However, prolonged ultrasonic treatment may have caused the rupture of glycosidic bonds due to excessive mechanical impact, resulting in partial polysaccharide degradation and a decline in the polysaccharide content (Hu et al., 2022). Thus, the optimal ultrasonic duration for ultrasound-assisted enzymatic extraction was determined to be 25 min.

#### 3.1.3 Effect of ultrasonic temperature

Fig. 1C shows how ultrasonic temperatures ranging from 25 °C to 65 °C affected the yield of SBP. Initially, the polysaccharide extraction rate rose with an increase in the temperature, peaking at 23.43 ± 0.10 % when the temperature was 45 °C, before decreasing thereafter. A rise in temperature reduces solution viscosity, enhances solvent mobility, accelerates molecular movement, and promotes the diffusion of active components (Fu, Wang, Belwal, Xie, et al., 2021). This is favorable for the release of active components from plant tissues and the dissolution of target molecules. Nevertheless, when the extraction temperature surpasses a specific threshold, the molecular structure of polysaccharides can be disrupted, leading to decomposition and ultimately impeding the extraction process (Zhang, Ye, et al., 2022). Thus, the ideal temperature for ultrasonic extraction was determined to be 45 °C.

#### 3.1.4 Effect of compound enzyme concentration

The extraction efficiency of SBP was significantly influenced by the amount of compound enzymes added. When the amount of compound enzyme increased from 2000 to 10,000 U/100 mL (Fig. 1D), the polysaccharide extraction rate first increased before decreasing thereafter, peaking at 23.023 ± 0.11 % after the addition of 6000 U/100 mL compound enzyme. Higher compound enzyme concentrations likely enhanced the interaction of cellulase and pectinase with the cell walls of the sea buckthorn tissues, increasing membrane permeability, reducing mass transfer resistance, and enhancing the diffusion coefficient, thus making polysaccharides more extractable. However, the excess addition of these enzymes may have led to over-saturation, preventing some enzyme molecules from interacting with sea buckthorn cells. Additionally, excessive enzymes and hydrolysis products could enshroud the sea buckthorn particles, impeding enzyme–sample interaction. Furthermore, the excessive enzymes could hydrolyze the glycosidic bonds of polysaccharides, leading to a significant drop in polysaccharide content (Lin et al., 2023). Accordingly, 6000 U/100 mL was identified as the optimal amount of enzyme for ultrasound-assisted enzymatic extraction.

#### 3.1.5 Effect of hydrolysis time

The impact of hydrolysis time on SBP yield is depicted in Fig. 1E. The polysaccharide extraction rate rose from 20.71 ± 0.14 % to 23.20 ± 0.05 % when the hydrolysis time increased from 70 min to 90 min. However, as the duration of enzymatic hydrolysis was extended to 110 min, the polysaccharide yield decreased dramatically to 22.07 ± 0.10 %. This was likely due to the structural changes and partial degradation of polysaccharides caused by prolonged hydrolysis. This phenomenon has been reported previously (Chai et al., 2019; Gu et al., 2023). Thus, the optimal hydrolysis time was determined to be 90 min.

#### 3.1.6 Effect of ultrasonic power

The impact of ultrasonic power on SBP yield is depicted in Fig. 1F. The polysaccharide yield progressively increased as the power increased from 280 W to 350 W, peaking at 350 W (i.e., 23.66 ± 0.18 %). Beyond this point, with a further increase in ultrasonic power, the yield of SBP progressively dropped to 22.66 ± 0.09 %. Ultrasonication induces cavitation bubbles in solvents. Upon collapsing, these cavitation bubbles generate high shear forces and micro-jets that enhance solvent permeability, facilitating the release of active cellular components (Huang et al., 2024). Nevertheless, excessive ultrasonic power can lead to an overabundance of cavitation bubbles, thereby reducing the efficiency of ultrasonic energy transmission (Hu et al., 2022). Moreover, high ultrasonic power negatively impacts the mobility and stability of polysaccharide molecules, causing structural damage and a consequent decrease in extraction yields (Lv et al., 2024). Thus, 350 W was identified as the optimal ultrasonic power for SBP extraction.

### 3.2 Response surface optimization for SBP extraction via ultrasound-assisted enzymatic treatment

#### 3.2.1 Data analysis and model fitting

Response surface methodology was applied to further enhance the SBP extraction process, as explained in section 2.2.4. The analysis of 29 sets of experimental data (Table 2) yielded the following second-order polynomial regression equation:

$$Y=23.40+0.69\;A–0.08B+0.53C–0.27D–0.14\mathrm{AB}+0.53\;\mathrm{AC}+1.00\;\mathrm{CE}+0.18\;\mathrm{BCE}+0.54\mathrm{BD}+0.16\mathrm{CD}–1.67A^{2}–0.85B^{2}–0.39C^{2}–1.93D^{2}$$

**Table 2:** SBP extraction rates obtained using the factor-level Box–Behnken response surface design.

Run	Solid-liquid ratio(g/mL)	Ultrasonic time(min)	Ultrasonic temperaturer(°C)	Enzyme concentration(U/100 mL)	SBP extraction yield(%)
1	35	25	55	6000	23.32
2	30	25	45	6000	23.32
3	30	30	55	6000	22.72
4	30	20	45	4000	21.84
5	35	30	45	6000	21.66
6	30	30	45	4000	20.57
7	25	25	45	4000	20.49
8	30	25	35	4000	21.03
9	25	25	45	8000	18.49
10	25	25	55	6000	20.62
11	30	25	45	6000	23.54
12	30	20	55	6000	22.58
13	30	30	35	6000	21.31
14	35	20	45	6000	21.99
15	30	30	45	8000	20.55
16	35	25	45	4000	19.05
17	30	25	45	6000	23.05
18	25	25	35	6000	20.48
19	30	20	35	6000	21.89
20	35	25	45	8000	21.05
21	35	25	35	6000	21.08
22	30	25	55	8000	21.43
23	30	25	45	6000	23.49
24	30	25	35	8000	20.15
25	25	20	45	6000	19.81
26	30	25	45	6000	23.59
27	25	30	45	6000	20.03
28	30	25	55	4000	21.66
29	30	20	45	8000	19.68

The outcomes of the model's variance analysis are shown in Table 3. Table 3 illustrates that the response surface model had an excellent fit and was highly significant, as 96.59 % of the predicted findings were within the experimental range (R^2^ = 0.9659, *p* < 0.0001, and *F* = 28.31). Furthermore, the lack-of-fit *p*-value was 0.1201; as this value was greater than 0.05, it was not significant. Additionally, the adjusted R^2^ (R^2^_adj_) value was 0.9318, and the predicted R^2^ (R^2^_pre_) value was 0.8182, showing a difference of less than 0.2. The coefficient of variation (CV) was 1.71 %, indicating minimal interference caused by confounding factors. The yield of SBP was significantly affected by the factors A, C, D, AD, A^2^, B^2^, and D^2^ (*p* < 0.01); additionally, AC, BD, and C^2^ also had notable impacts on SBP yield (*p* < 0.05). According to the *F*-values, the order of influence exerted by the factors on the polysaccharide yield was as follows: A > C > D > B; AD > BD > AC > BC > CD > AB.

**Table 3:** ANOVA for the quadratic polynomial model for SBP extraction yield.

Source	Sum of Squares	df	Mean Square	F-Value	P-valuea	Significance
Model	53.00	14	3.79	28.31	< 0.0001	significant
A	5.64	1	5.64	42.21	< 0.0001	**
B	0.0752	1	0.0752	0.5624	0.4657	
C	3.40	1	3.40	25.45	0.0002	**
D	0.9020	1	0.9020	6.75	0.0211	*
AB	0.0756	1	0.0756	0.5655	0.4645	
AC	1.10	1	1.10	8.24	0.0123	*
AD	4.00	1	4.00	29.91	< 0.0001	**
BC	0.1296	1	0.1296	0.9692	0.3416	
BD	1.14	1	1.14	8.56	0.0111	*
CD	0.1056	1	0.1056	0.7899	0.3892	
A^2^	18.06	1	18.06	135.05	< 0.0001	**
B^2^	4.67	1	4.67	34.93	< 0.0001	**
C^2^	1.00	1	1.00	7.51	0.0159	*
D^2^	24.13	1	24.13	180.42	< 0.0001	**
Residual	1.87	14	0.1337			
Lack of Fit	1.68	10	0.1679	3.49	0.1201	not significant
Pure Error	0.1927	4	0.0482			
Cor Total	54.87	28				
R^2^	0.9659		Adeq precision	21.1303		
Adj-R^2^	0.9318		Std. Dev.	0.3657		
Pred-R^2^	0.8182		C.V. %	1.71		

Note: * represents *p* < 0.05; ** represents *p* < 0.01.

#### 3.2.2 Response surface analysis

**Fig. 2:**
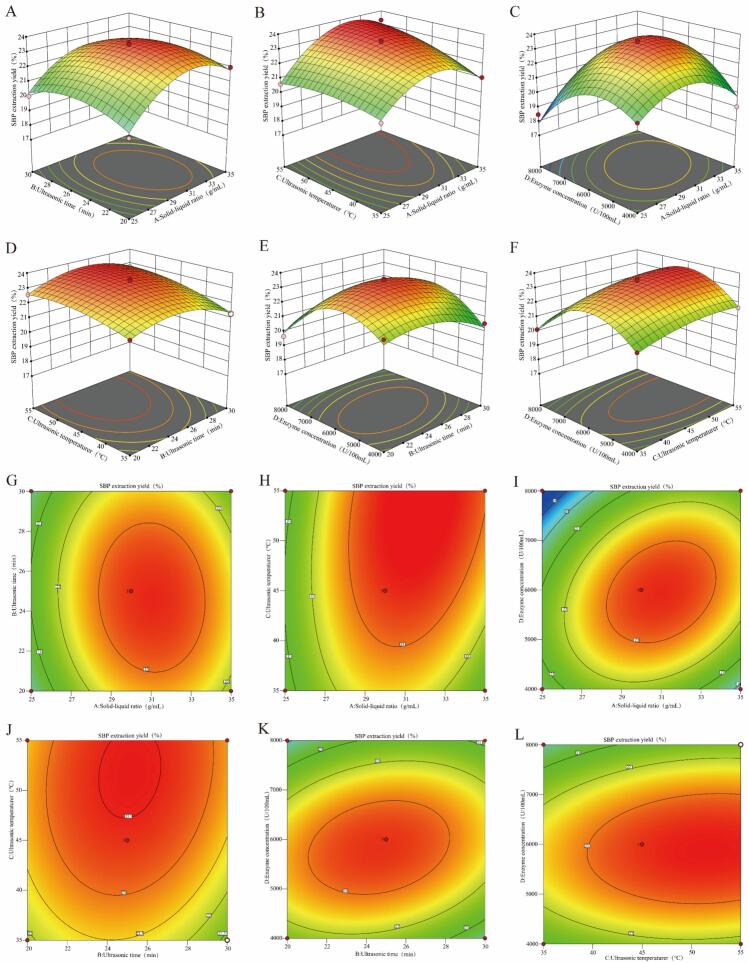
Response surface analysis results. Response surface plots (A-E) and contour plots (G-L) showing the effects of various parameters on the extraction yield of SBP.

The three-dimensional (Fig. 2A-F) and two-dimensional (Fig. 2G-L) response surface plots prepared using Design-Expert 13.0.0 software are shown in Fig. 2. When the annular region of the two-dimensional response surface plot approaches an elliptical shape and the contour lines become denser, and the three-dimensional response surface plot becomes steeper, the plot suggests a strong interaction effect of two factors on the efficiency of extraction and a higher sensitivity of response values to changes in extraction parameters (Lv et al., 2024).

As illustrated in Fig. 2A-C, among the four factors, the solid–liquid ratio caused the steepest slope in the response surface, indicating that this factor had the strongest influence on SBP extraction. Fig. 2D and E show that among the other three factors, ultrasonic duration had the weakest effect on SBP extraction. Fig. 2F clearly demonstrates that the effect of ultrasonic temperature on SBP extraction was stronger than that of compound enzyme concentration. The yield of SBP was therefore primarily determined by the solid–liquid ratio (A), followed by the ultrasonic temperature (C), the amount of composite enzyme added (D), and the ultrasonic duration (B). These results were consistent with the findings of polynomial variance analysis, as shown in Table 3.

Additionally, a thorough examination of the elliptical rings in contour plots (Fig. 2G-L) and the surface steepness (Fig. 2A-C) revealed that A (solid–liquid ratio) and D (compound enzyme concentration), A (solid–liquid ratio) and B (enzymatic hydrolysis time), and B (enzymatic hydrolysis time) and D (compound enzyme concentration) induced a large surface slope and curvature. The interaction between these factors was significant and had a great influence on the extraction rate of SBP (*p* < 0.05). The CD, BC, and AC interactions had no significant effects on the extraction yield of SBP. These results were consistent with the findings of variance analysis (Table 3).

#### 3.2.3 Optimization and validation of SBP extraction

The ideal processing parameters for SBP extraction were calculated using Design Expert software. These parameters were as follows: 1:31.96 (g/mL) for the solid–liquid ratio, 26.16 min for the ultrasonic duration, 51.79 °C for the ultrasonic temperature, and 5986.41 U/100 mL for the amount of compound enzyme. Under these conditions, the maximum theoretical extraction rate of SBP was 23.68 %. For experimental validation, the ideal extraction conditions were adjusted for practical reasons and convenience of use, as follows: solid–liquid ratio of 1:32 (g/mL), ultrasonic period of 26 min, ultrasonic temperature of 52 °C, and compound enzyme concentration of 6000 U/100 mL. The SBP extraction rate under these modified conditions was 24.07 ± 0.15 %, with a relative error of 1.6 % when compared to the expected value. These findings demonstrated the viability of the Box–Behnken optimization model.

### 3.3 Analysis of purified SBP fractions

**Fig. 3:**
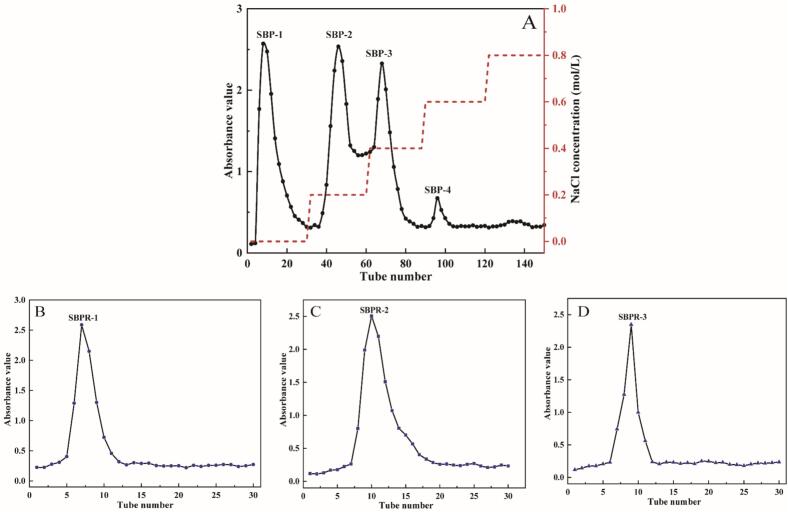
Elution curves of SBP (A) obtained via DEAE-52 NaCl concentration gradient elution (0, 0.2, 0.4, 0.6 and 0.8 M) and SBPR-1 (B), SBPR-2 (C), and SBPR-3 (D) obtained via Sephadex G-150 distilled water elution.

The separation of SBP fractions was performed using a DEAE-52 chromatography column, as shown in Fig. 3A. In total, four fractions eluted with deionized water and 0.2 M, 0.4 M, and 0.6 M NaCl were obtained. These fractions were designated SBP-1, SBP-2, SBP-3, and SBP-4. SBP-1 was found to be a neutral polysaccharide, while SBP-2, SBP-3, and SBP-4 appeared to be acidic polysaccharides. Due to the low content of SBP-4 and the absence of an absorption peak upon 0.8 M NaCl elution, only SBP-1, SBP-2, and SBP-3 were further purified via Sephadex G-150 gel column chromatography (Fig. 3B-D). Each eluate showed a single symmetrical peak, corresponding to SBPR-1, SBPR-2, and SBPR-3, and the total sugar contents in these fractions were 84.52 %, 87.76 %, and 92.98 %, respectively. The protein contents of SBPR-1, SBPR-2, and SBPR-3 were low at 0.34 %, 0.25 %, and 0.13 %, and the uronic acid contents were 0.18 %, 1.31 %, and 2.52 %, respectively. This indicated that the uronic acid content in the acidic polysaccharides was higher than that in the neutral polysaccharide.

### 3.4 Monosaccharide composition of SBPRs

**Fig. 4:**
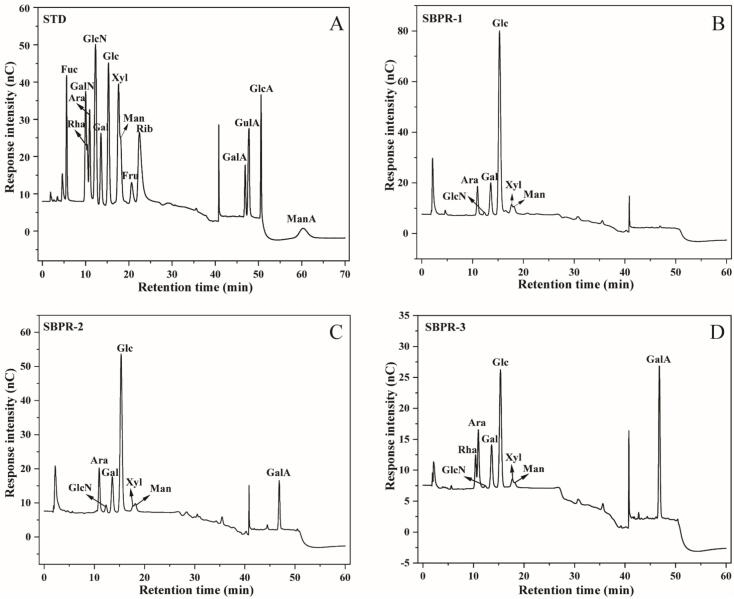
Monosaccharide composition of SBPRs. Ion chromatograms of the STD (mixed standard 15 sugar), SBPR-1, SBPR-2, and SBPR-3.

Monosaccharide composition analysis is crucial for the structural evaluation of polysaccharides. Differences in monosaccharide types and contents affect the types, linkage, and spatial configurations of glycosidic bonds in polysaccharides, thus influencing their biological activity. Through comparisons with monosaccharide standards, the monosaccharide compositions of SBPR-1, SBPR-2, and SBPR-3 were ascertained using HPAEC-PAD. The outcomes, displayed in Table 4 and Fig. 4, indicated that SBP mainly consisted of rhamnose, arabinose, glucosamine hydrochloride, galactose, glucose, xylose, mannose, and glucuronic acid. The monosaccharide composition ratios varied significantly among the three fractions of SBP. Specifically, SBPR-1 mainly contained glucose (59.1 %), galactose (20.7 %), and arabinose (10.5 %), but no glucuronic acid was detected in this fraction, indicating that it was a neutral polysaccharide. In SBPR-2, the content of glucose (35.9 %) and galactose (17.2 %) was significantly lower, although these monosaccharides remained predominant. Moreover, SBPR-2 contained a higher content of arabinose (11.8 %) as well as a substantial amount of glucuronic acid (27.9 %), and it was thus classified as an acidic polysaccharide. SBPR-3, another acidic polysaccharide, exhibited the highest glucuronic acid content (49.8 %), with significantly lower amounts of glucose (14.3 %), galactose (11.0 %), and rhamnose (12.7 %).

**Table 4:** Monosaccharide molar ratios in SBPR-1, SBPR-2, and SBPR-3.

Factions	Rha	Ara	GlcN	Gal	Glc	Xyl	Man	GalA
SBPR-1	–	10.5	0.5	20.7	59.1	3.3	5.9	–
SBPR-2	–	11.8	0.9	17.2	35.9	1.4	5.2	27.9
SBPR-3	12.7	9.4	0.1	11.0	14.3	1.3	1.4	49.8

Additionally, the uronic acid content was the highest in SBPR-3, followed by SBPR-2 and SBPR-1, consistent with the results of chemical analysis (section 3.3). SBPR-1 and SBPR-2 were identified as neutral and acidic polysaccharides primarily composed of glucose, while SBPR-3 was an acidic polysaccharide that mainly contained glucuronic acid and glucose. Bao et al. (Bao et al., 2024) reported that SBP are composed of arabinose, xylose, mannose, glucose, and galactose, while Xie (Xie et al., 2023) concluded that they consist of galactosamine, galactose, arabinose, glucose, mannose, and xylose. The findings of the present study are in line with their results. The the variance in monosaccharide types could be attributed to differences in extraction methodologies across these studies.

### 3.5 Molecular weight of SBPRs

The molecular weight of the polysaccharides was determined using HPGPC. With the retention time (Rt) on the X-axis, and lgMp-RT (peak molecular weight), lgMw-RT (weight average molecular weight), and lgMn-RT (number average molecular weight) on the Y-axis, the regression equation lgMp-RT was obtained as follows: y = −0.2039× + 11.861 (R^2^ = 0.9938); lgMw-RT: y = −0.2055× + 11.913 (R^2^ = 0.9939); lgMn-RT: y = −0.2055× + 11.906 (R^2^ = 0.9933).

**Fig. 5:**
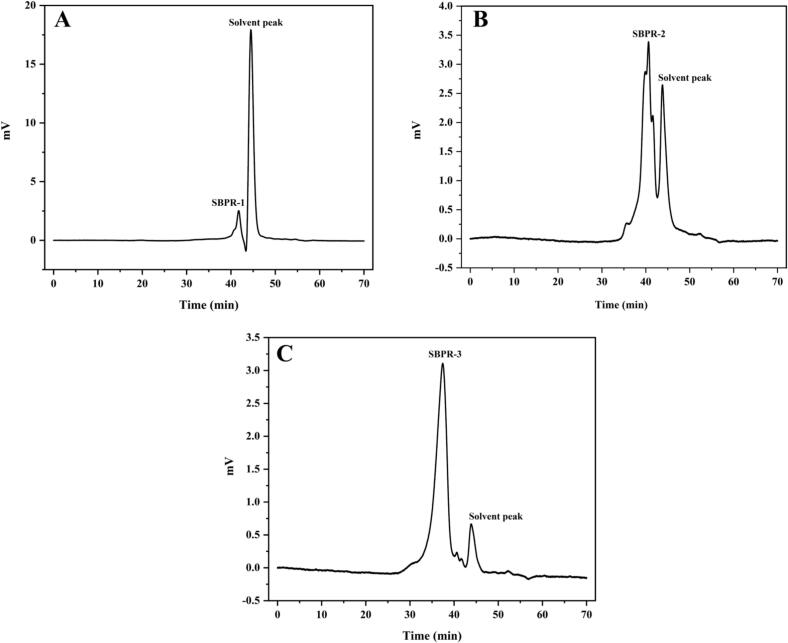
Molecular weight distribution of SBPR-1, SBPR-2, and SBPR-3.

Fig. 5. shows the molecular weight distribution of the SBPRs. SBPR-1 and SBPR-2 appeared to be relatively uniform polysaccharides with molecular weights of 2256 Da and 17,457 Da, respectively. Meanwhile, the molecular weight distribution of SBPR-2 was more complex, consisting of components sized 3849, 5479 and 2402 Da.

### 3.6 FT-IR spectral analysis

**Fig. 6:**
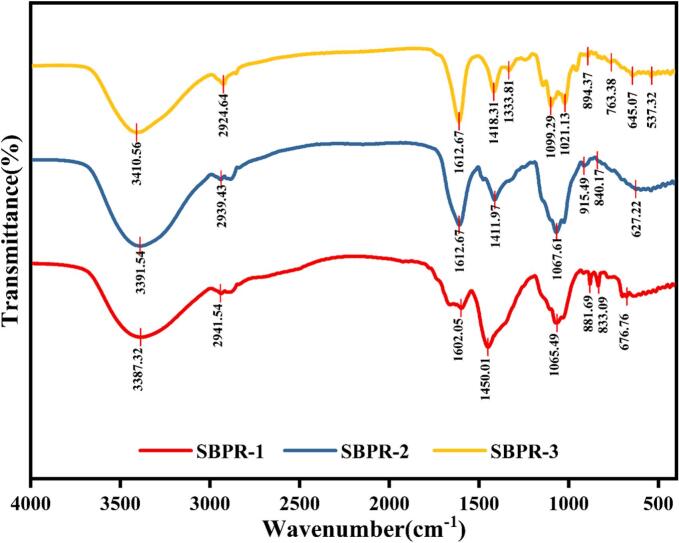
FT-IR spectra of SBPR-1, SBPR-2, and SBPR-3 at within the range of 500–4000 nm.

The FT-IR analysis of SBPR-1, SBPR-2, and SBPR-3 was conducted to compare their glycosidic bond types, functional groups, and glycan ring configurations (Fig. 6.). All fractions exhibited typical plant polysaccharide absorption peaks within the 4000–400 cm^−1^ range. The broad absorption peaks at 3387.32 cm^−1^ (SBPR-1), 3391.54 cm^−1^ (SBPR-2), and 3410.56 cm^−1^ (SBPR-3) corresponded to the O—H stretching vibrations of sugar residues. Meanwhile, the weak absorption peaks near 2940 cm^−1^ were indicative of asymmetric C—H stretching vibrations, possibly due to -CH_2_ or -CH_3_ groups (Ji et al., 2020), which are characteristic of polysaccharides. Since asymmetric C=O stretching vibrations typically occur near 1600–1650 cm^−1^, the peaks at 1602.05 cm^−1^ (SBPR-1) and 1612.67 cm^−1^ (SBPR-2, SBPR-3) were attributed to the asymmetric stretching vibrations of the -COOH group (Jiao et al., 2024). The absorption peaks around 1411.97–1450.01 cm^−1^ corresponded to the symmetric stretching vibrations of carboxylate anions (COO-), representing non-esterified carboxyl groups that likely exist as carboxylate ions (Košťálová & Hromádková, 2019), potentially involving the N—H stretching vibrations in proteins (Zhang et al., 2022). Peaks near 800–1000 cm^−1^ were attributed to pyranose ring vibrations. Moreover, strong, broad peaks at 1056.49 cm^−1^ (SBPR-1), 1067.61 cm^−1^ (SBPR-2), and 1099.29 cm^−1^ (SBPR-3), indicative of C-O-C and C-O-H stretching vibrations were also observed, demonstrating that all three fractions contained pyranose-type polysaccharides (Gu et al., 2023). SBPR-3 showed a signal near 1250 cm^−1^, which was attributed to the stretching vibrations of S=O, suggesting the existence of potential sulfate groups in this fraction (Bai et al., 2020). The typical absorption peaks at 900 cm^−1^ and 840 cm^−1^ corresponded to β-glycosidic and α-glycosidic linkages, respectively. Meanwhile, SBPR-1 and SBPR-2 were found to contain α-configuration polysaccharides, while all fractions contained β-configuration polysaccharides.

### 3.7 SEM analysis

**Fig. 7:**
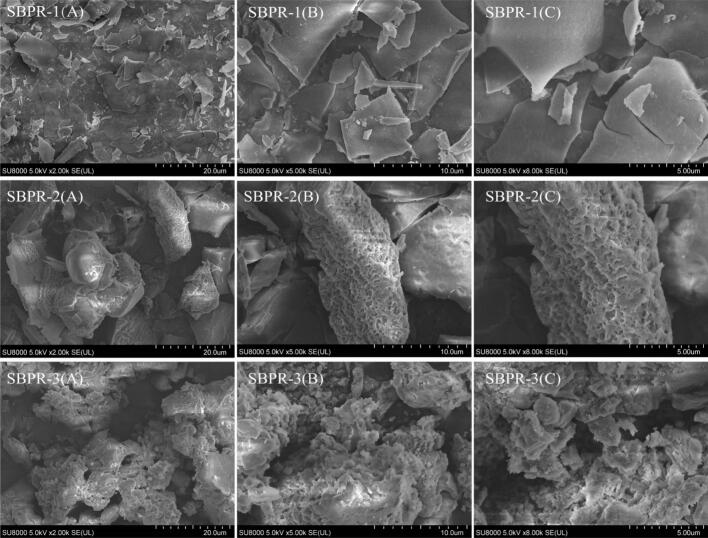
SEM images of SBPR-1, SBPR-2, and SBPR-3 at 2000×, 5000×, and 8000 × .

SEM was performed to observe the surface microstructure of SBPR-1, SBPR-2, and SBPR-3. The SEM images of SBPR-1, SBPR-2, and SBPR-3 at magnifications of 2000×, 5000×, and 8000× are shown in Fig. 7. Notably, SEM revealed clear distinctions in the surface morphologies of the SBPRs. SBPR-1 exhibited a sheet-like structure, with a smooth, flat surface and relatively high density and aggregation. In contrast, SBPR-2 showed irregular, cone-like aggregation, along with some ultrasound-induced cavities, a rough surface, and dense pores. Meanwhile, the surface of SBPR-3 had a loosely agglomerated flocculent structure, with numerous ultrasound-induced cavities and an uneven surface. The differences in the surface structures of SBPRs could be attributed to the differences in the eluents, which alter the binding strength of polysaccharide aggregates and affect intermolecular interactions (Rozi et al., 2019). Additionally, freeze-drying could affect the structure of these polysaccharides, and the variations in the monosaccharide compositions, glycosidic linkages, and uronic acid contents of the three components could also contribute to the distinct morphological structures (Romdhane et al., 2017).

### 3.8 Thermal stability analysis

**Fig. 8:**
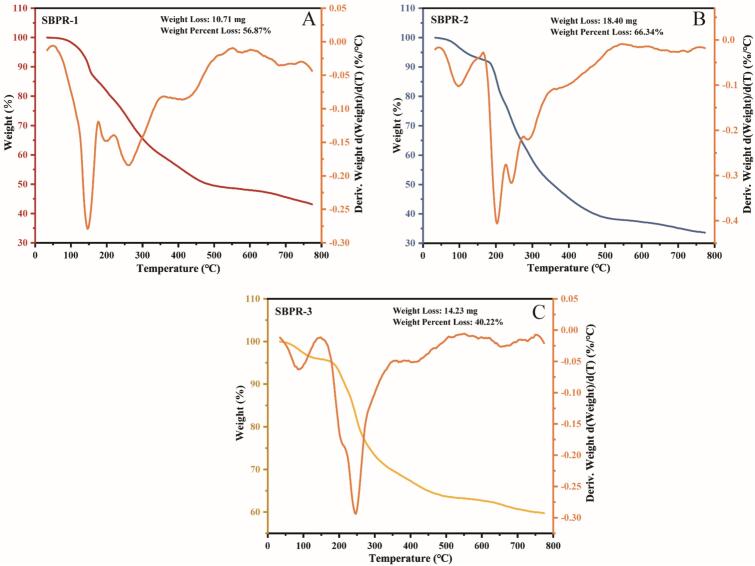
TG and DTG curves of SBPR-1, SBPR-2, and SBPR-3 at 0–800 °C.

The processing and use of polysaccharides in the food and pharmaceutical industries depend on their thermal stability. The effect of heat treatment on mass is measured using thermogravimetric analysis (TGA), which can reveal the oxidation, breakdown, and dehydration of polysaccharides across time and temperature fluctuations (Chen et al., 2020). Fig. 8. displays the thermogravimetric (TG) and differential thermogravimetric (DTG) curves for SBPR-1, SBPR-2, and SBPR-3. In the present study, the results showed that when the temperature rose, the mass of the three polysaccharide components decreased. According to the TG curves, within the range of 35–800 °C, the polysaccharides primarily underwent three stages of degradation. The evaporation of both bound and free water in the polysaccharide samples was believed to drive the first step of mass loss, which occurred between 35 °C and 160 °C. The second step (180–500 °C) was attributed to the cleavage and depolymerization of the molecules in the sample, including the breakage of C—C and C—O bonds, as well as the loss of crystalline water (Fang et al., 2020). As the temperature rose to 775 °C, the mass loss rate of the three components decreased gradually. The final proportion of residual polysaccharide mass in the samples was as follows: SBPR-3 (59.78 %) > SBPR-1 (43.13 %) > SBPR-2 (33.66 %). SBPR-2 showed the highest mass loss rate (66.34 %), while SBPR-1 had the lowest mass loss rate (40.22 %). DTG curves demonstrated that the fastest weight loss in SBPR-1, SBPR-2, and SBPR-3 occurred at temperatures of 150.1 °C, 204.0 °C, and 245.7 °C, respectively, with weight loss rates of 3.92 %/min, 5.35 %/min, and 3.47 %/min. Thus, SBPR-3 exhibited relatively optimal thermal stability, whereas SBPR-1 demonstrated the least thermal stability. However, all three polysaccharide components showed good overall thermal stability and similar thermodynamic curve trends. The variations in the monosaccharide compositions, chemical constituents, and structures of the three components may have led to some differences in heat stability and degradation properties.

### 3.9 Analysis of antioxidant activity

**Fig. 9:**
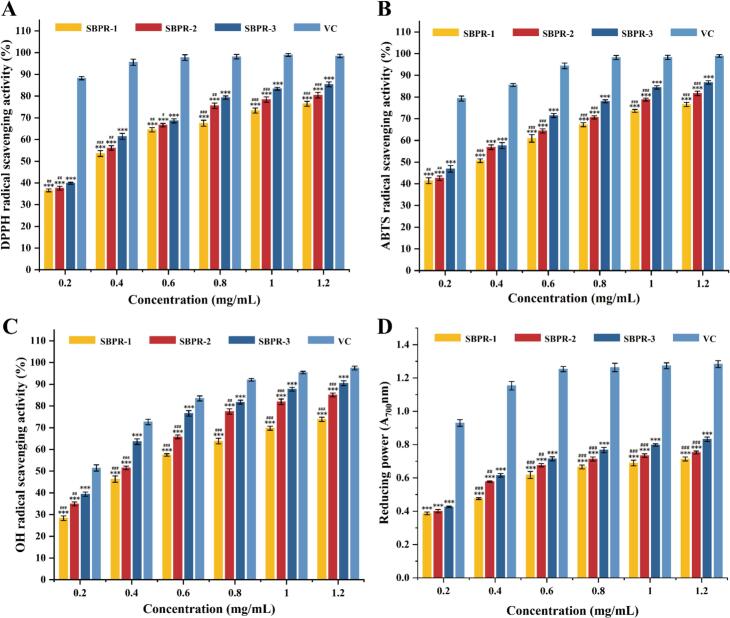
In vitro antioxidant activity of SBPs at concentrations of 0.2–1.2 mg/mL. (A) DPPH radical scavenging; (B) ABTS radical scavenging; (C) hydroxyl radical scavenging; (D) Total reducing power (Compared with VC: ^⁎⁎⁎^*p* < 0.001, ^⁎⁎^*p* < 0.01; Compared with SBPR-3: ^**###**^*p* < 0.001，^**##**^*p* < 0.01).

DPPH, a stable nitrogen-centered free radical with lipophilic properties, is commonly employed for evaluating the antioxidant capacity of substances in vitro (Fang et al., 2020). As depicted in Fig. 9A, the DPPH radical scavenging activity of the three SBPR components increased progressively with their concentration (0.2–1.2 mg/mL), showing a dose-dependent effect. SBPR-3 demonstrated the highest capacity to scavenge DPPH radicals across all concentrations, achieving an 85.42 ± 1.12 % scavenging rate at a polysaccharide concentration of 1.2 mg/mL. SBPR-2 and SBPR-1 came in second and third, respectively, with scavenging rates of 80.45 ± 1.28 % and 76.48 ± 1.10 %. The IC_50_ value against DPPH was the highest for SBPR-1 (0.34 mg/mL), followed by SBPR-2 (0.32 mg/mL) and SBPR-3 (0.28 mg/mL). Given that lower IC_50_ values indicate a higher free radical scavenging ability, these findings demonstrated that SBPR-3 had the strongest scavenging ability for DPPH radicals.

ABTS can be oxidized by reagents such as potassium persulfate and hydrogen peroxide. However, stable blue-green ABTS·^+^ radicals become colorless in the presence of antioxidants. Hence, the ABTS method is used to assess the antioxidant capacity of plant extracts, pure substances, and beverages (He et al., 2018). As shown in Fig. 9B, all three polysaccharide components showed excellent ABTS radical scavenging effects. The IC_50_ value was the highest for SBPR-1 (0.32 mg/mL), followed by SBPR-2 (0.29 mg/mL) and SBPR-3 (0.25 mg/mL). The strongest ABTS radical scavenging rate of 86.71 ± 0.76 % was achieved by SBPR-3 at a polysaccharide concentration of 1.2 mg/mL. The radical scavenging performance of all three SBPRs was also concentration-dependent. The capacity to scavenge ABTS radicals at this concentration was the highest in SBPR-3 (86.71 ± 0.76 %), followed by SBPR-2 (81.65 ± 1.03 %) and SBPR-1 (76.58 ± 0.96 %). However, all the polysaccharide components showed a weaker scavenging ability than equivalent concentrations of vitamin C (VC).

Hydroxyl radicals are critical reactive oxygen species and have a high electron affinity. Thus, an excess of these radicals can induce oxidative damage, leading to various disorders (Tang et al., 2023). The capacity of the three SBP components to scavenge hydroxyl radicals increased with their concentration, as shown in Fig. 9C, demonstrating a dose–response relationship. The IC_50_ value was the highest in SBPR-1 (0.46 mg/mL), followed by SBPR-2 (0.34 mg/mL) and SBPR-3 (0.27 mg/mL). All components showed the strongest scavenging capacities at a concentration of 1.2 mg/mL, with SBPR-3 demonstrating the highest antioxidant activity (90.48 ± 1.10 %), followed by SBPR-2 (85.08 ± 0.87 %) and SBPR-1 (73.87 ± 0.96 %). These capacities were all over 50 % of the scavenging capacity (97.49 ± 0.90 %) of VC, with SBPR-3 achieving a hydroxyl scavenging rate comparable to that of VC (92.82 %).

When assessing a substance's antioxidant capacity, its total reducing power is also a crucial metric to consider. Reducing agents can release hydrogen atoms, inhibiting peroxide formation by breaking free radical chains. As shown in Fig. 9D, the total reducing capacity of SBP and VC at different concentrations indicated that the polysaccharide components possessed excellent reducing power. Their reducing power was positively correlated with the concentration in the 0.2–1.2 mg/mL range. However, the total reducing power began to stabilize at a concentration of 0.6 mg/mL. The total reducing capacities of the polysaccharides at a concentrations of 1.2 mg/mL were as follows: SBPR-3 (0.83 ± 0.01) > SBPR-2 (0.75 ± 0.01) > SBPR-1 (0.71 ± 0.01). Although the reducing power of the SBP components was lower than that of VC, their antioxidant capabilities remained substantial.

Based on these results and analyses, it was evident that SBPR-3 had higher antioxidant activity than SBPR-2 and SBPR-1, with the acidic polysaccharides (SBPR-3 and SBPR-2) demonstrating superior antioxidant activities than the neutral polysaccharide (SBPR-1). Polysaccharides contain hydroxyl and aldehyde groups, which have reducing properties. Moreover, monosaccharides provide hydrogen ions that combine with free radicals to generate more stable radicals. Our results suggested that SBPR-3 possesses a better hydrogen-donating capacity, which contributes to its superior antioxidant ability. Additionally, the structure and chemical composition of polysaccharides are known to affect their antioxidant action (Fang et al., 2020). The presence of -COOH and -OH and the high content of uronic acid and sugars in the acidic polysaccharide SBPR-3 were speculated to enhance its antioxidant properties (Zhang et al., 2023).

## 4 Conclusion

In this study, SBP were extracted using an ultrasonic-assisted enzymatic treatment method. The process of SBP extraction was optimized using single-factor experiments and response surface methodology. The optimal conditions were found to be as follows: a solid–liquid ratio of 1:32 (g/mL), ultrasonic duration of 26 min, ultrasonic temperature of 52 °C, and composite enzyme concentration of 6000 U/100 mL. The maximum extraction yield of SBP reached 24.07 ± 0.15 %Subsequently, SBP were purified using DEAE-52 cellulose and Sephadex G-150 gel columns, yielding one neutral polysaccharide component, SBPR-1, and two acidic polysaccharide components, SBPR-2 and SBPR-3. The SBPRs contained different molar ratios of Rha, Ara, GlcN, Gal, Glc, Xyl, Man and GalA. The molecular weights of SBPR-1 and SBPR-3 were found to be 2256 Da and 17,457 Da, respectively. Moreover, SBPR-2 was mainly composed of units sized 3849, 5479 and 2402 Da.SBPR-1 and SBPR-2 had both α- and β-configured polysaccharides, whereas SBPR-3 contained only β-configured polysaccharides. Additionally, the surface morphology of the SBPRs exhibited significant differences. Nevertheless, the SBPRs showed good thermal stability. The SBPRs also exhibited a high scavenging capability for DPPH, ABTS, and hydroxyl radicals, while also possessing substantial reducing power. Furthermore, the antioxidant capacity of the acidic polysaccharides surpassed that of the neutral polysaccharide. Overall, the findings showed that ultrasonic-assisted compound enzyme extraction is an environmentally friendly and efficient technique for extracting SBP. This present study provides a theoretical scientific basis for the advanced processing and utilization of sea buckthorn berries. Nevertheless, further research is required for explore the specific structural characterization of SBP and the evaluation of SBP bioactivity.

## CRediT authorship contribution statement

**Jing Wen:** Writing – original draft, Methodology, Data curation, Conceptualization. **Ruijie Huang:** Writing – review & editing, Supervision. **Shi Li:** Writing – review & editing, Methodology. **Lin Jiang:** Investigation, Data curation. **Liheng Shao:** Visualization, Data curation. **Qin Zhang:** Writing – review & editing, Supervision, Funding acquisition. **Chunhui Shan:** Writing – review & editing, Supervision, Methodology, Funding acquisition, Conceptualization.

## Declaration of competing interest

The authors declare that they have no known competing financial interests or personal relationships that could have appeared to influence the work reported in this paper.

## Data Availability

Data will be made available on request.
